# The Vitamin K-Dependent Anticoagulant Factor, Protein S, Regulates Vascular Permeability

**DOI:** 10.3390/cimb46040205

**Published:** 2024-04-09

**Authors:** Aurélie Joussaume, Chryso Kanthou, Olivier E. Pardo, Lucie Karayan-Tapon, Omar Benzakour, Fatima Dkhissi

**Affiliations:** 1 Université de Poitiers, CHU de Poitiers, ProDiCeT, UR 24144 Poitiers, France; joussaumeaurelienc@gmail.com (A.J.); omar.benzakour@univ-poitiers.fr (O.B.); 2 Division of Clinical Medicine, School of Medicine & Population Health, University of Sheffield, Sheffield S10 2RX, UK; c.kanthou@sheffield.ac.uk; 3 Division of Cancer, Department of Surgery and Cancer, Imperial College London, London SW7 2AZ, UK; o.pardo@imperial.ac.uk; 4 Université de Poitiers, CHU de Poitiers, ProDiCeT, Laboratoire de Cancérologie Biologique, UR 24144 Poitiers, France; lucie.karayan-tapon@chu-poitiers.fr

**Keywords:** protein S, TAM receptors, vascular permeability, endothelial cell biology, vascular endothelial cadherin, actin remodeling

## Abstract

Protein S (PROS1) is a vitamin K-dependent anticoagulant factor, which also acts as an agonist for the TYRO3, AXL, and MERTK (TAM) tyrosine kinase receptors. PROS1 is produced by the endothelium which also expresses TAM receptors, but little is known about its effects on vascular function and permeability. Transwell permeability assays as well as Western blotting and immunostaining analysis were used to monitor the possible effects of PROS1 on both endothelial cell permeability and on the phosphorylation state of specific signaling proteins. We show that human PROS1, at its circulating concentrations, substantially increases both the basal and VEGFA-induced permeability of endothelial cell (EC) monolayers. PROS1 induces p38 MAPK (Mitogen Activated Protein Kinase), Rho/ROCK (Rho-associated protein kinase) pathway activation, and actin filament remodeling, as well as substantial changes in Vascular Endothelial Cadherin (VEC) distribution and its phosphorylation on Ser^665^ and Tyr^685^. It also mediates c-Src and PAK-1 (p21-activated kinase 1) phosphorylation on Tyr^416^ and Ser^144^, respectively. Exposure of EC to human PROS1 induces VEC internalization as well as its cleavage into a released fragment of 100 kDa and an intracellular fragment of 35 kDa. Using anti-TAM neutralizing antibodies, we demonstrate that PROS1-induced VEC and c-Src phosphorylation are mediated by both the MERTK and TYRO3 receptors but do not involve the AXL receptor. MERTK and TYRO3 receptors are also responsible for mediating PROS1-induced MLC (Myosin Light Chain) phosphorylation on a site targeted by the Rho/ROCK pathway. Our report provides evidence for the activation of the c-Src/VEC and Rho/ROCK/MLC pathways by PROS1 for the first time and points to a new role for PROS1 as an endogenous vascular permeabilizing factor.

## 1. Introduction

Protein S (PROS1), encoded by the *PROS1* gene, is a vitamin K-dependent plasma glycoprotein that acts as an inhibitor of blood coagulation either as a cofactor for or independently of activated protein C [1,2]. PROS1 circulates in human plasma at a concentration of ~25 μg/mL in free form (40%) and in complex (60%) with C4b-binding protein (C4BP) [3]. PROS1 is also produced at many extrahepatic sites, including macrophages [4], vascular endothelial cells (ECs) [5], and vascular smooth muscle cells [6,7]. In these cells, PROS1 plays no apparent role in blood coagulation, but acts as a ligand for TYRO3 and MERTK, two tyrosine kinase receptors of the TAM (TYRO3, AXL, and MERTK) family. PROS1 activates TYRO3 in brain endothelial cells [8] and neurons [9], MERTK in macrophages [10] and human umbilical vein endothelial cells [11], and both MERTK and TYRO3 in the retinal epithelium [12]. PROS1 activation of MERTK or TYRO3 exerts regulatory effects over a large repertoire of cellular activities, including cell proliferation, migration, and apoptotic cell clearance by phagocytosis [13,14,15,16,17,18,19].

Converging lines of evidence suggest that PROS1 may have crucial regulatory functions within the vasculature [20]. PROS1, as well as TYRO3 and MERTK, is expressed within the vasculature by ECs, pericytes [21], and vascular smooth muscle cells [6,20]. Heterozygous mutations or polymorphisms of the *PROS1* gene are associated with venous and arterial thrombosis [22,23]. PROS1 knock-out mice are not viable but die at embryonic stages owing to lethal thrombotic complications and vascular dysgenesis. An analysis of mice in which the *PROS1* gene was conditionally deleted in either vascular smooth muscle or endothelial cells revealed serious defects in vessel development and homeostasis and indicated that endothelial cells contribute to over 40% of circulating PROS1 [22].

The endothelial cells lining blood vessels play a central role in vascular barrier function. Impaired endothelial permeability is associated with various pathological states such as tumor angiogenesis and diabetic retinopathy [24,25]. Endothelial permeability is controlled by numerous extracellular stimuli that are either endogenously produced within the vasculature or endocrine molecules that are active within the blood stream [26]. The molecular mechanisms underlying endothelial permeability involve both cytoskeleton remodeling and intercellular adhesion [27]. Actin cytoskeleton remodeling is regulated by several signaling pathways, among which, the Rho/ROCK pathway that phosphorylates MLC and the p38 MAPK pathway that phosphorylates HSP27 (Heat Shock Protein 27), are the major ones [28,29]. Intercellular adhesion is mainly regulated by Vascular Endothelial Cadherin (VEC), as its extracellular domain enables intercellular adhesion while its cytoplasmic domain interacts with the actin cytoskeleton via α- and β-catenins and stabilizes cell–cell junctions [30].

Phosphorylation of VEC on residues of Ser^665^ or Tyr^685^ is associated with VEC dissociation from catenins followed by its internalization and with the cleavage of its extracellular domain by proteases (i.e., MMP-9 or ADAM12), respectively [31,32,33]. It has been demonstrated that VEGFR2 (Vascular Endothelial Growth Factor Receptor 2) activation leads to Ser^144^ autophosphorylation of p21-activated kinase-1 (PAK-1), a downstream Rac effector, which in turn phosphorylates VEC on Ser^665^, thereby triggering its internalization [34,35,36,37]. Moreover, several studies also provided evidence that VEGFR2 activation may lead to c-Src phosphorylation on Tyr^416^ which in turn phosphorylates VEC on Tyr^685^, thereby triggering its cleavage into 90–100 kDa N-terminal and 35 kDa C-terminal fragments. The former is released into the medium while the latter remains intracellular [38,39,40]. VEC internalization or cleavage weaken both cell–cell interactions and inter-endothelial junctions leading to increased endothelial permeability [35,38,39,40]. Although the endothelium expresses TAM receptors and is a major source of endogenously produced PROS1 within the vasculature [22], the pathophysiological relevance of PROS1’s interactions with ECs has not been fully characterized. In a previous study, we demonstrated that, in cultured ECs, human PROS1 inhibits multiple VEGFA- (Vascular Endothelial Growth Factor A) induced angiogenesis events [11]. In the present report, we studied the possible effects of human PROS1 on EC monolayer permeability. We provide clear evidence that human PROS1 increases endothelial monolayer permeability through the regulation of cytoskeleton remodeling as well as VEC phosphorylation, internalization, and cleavage. We suggest that, besides its known role as an inhibitor of blood clotting, human PROS1 is a key endogenous regulatory factor for endothelium permeability, and therefore central to homeostasis of the vascular system.

## 2. Materials and Methods

### 2.1. Cells

Human umbilical vein endothelial cells (HUVECs, Lonza Walkersville, Walkersville, MD, USA) were cultured in endothelial growth medium-2 (EGM2; cc-3162, Lonza Walkersville), which refers to a growth factor-containing medium that has been described previously [11], and used at early passages 1–4. When indicated, cultures were switched to EBM-2 (Endothelial Basal Medium 2) (Lonza Walkersville), which refers to a growth factor-depleted medium. Cells were kept at 37 °C in a 5% CO_2_ humidified incubator. General cell culture dishes and reagents were obtained from Thermo Fisher (Waltham, MA, USA).

### 2.2. Materials

Human PROS1 was from Enzyme Research Laboratories (Swansea, UK). It was purified from fresh frozen human plasma, characterized by the supplier as a single band at 69 kDa on SDS-PAGE (Sodium Dodecyl-Sulfate PolyAcrylamide Gel Electrophoresis) and described as highly pure. PROS1 was used in all experiments at 10 μg/mL. Recombinant human VEGFA 165 isoform was from Invitrogen (Waltham, MA, USA) and was used at 20 ng/mL.

### 2.3. Transwell Permeability Assay

This assay was conducted as described previously [41,42]. Fibronectin-coated transwell inserts of 3.0 µm pore size set in 24 well plates from Corning (Corning, NY, USA) were used. HUVECs were plated in 200 μL of complete medium at a density of 1.5 × 10^5^ cells/insert and cultured for 5 days with 700 μL of complete medium in the lower chamber. Cell cultures were then switched to EBM-2 basal medium at 100 μL in the upper and 500 μL in the lower chamber and exposed to PROS1 at 10 μg/mL and/or VEGFA at 20 ng/mL for the indicated duration. FITC-dextran (Fluorescein IsoThioCyanate coupled dextran, 40 kDa) from Sigma Aldrich (St. Louis, MI, USA) at 1 mg/mL was then added to the upper chamber for 30 min, then 100 μL of the culture media of the lower chamber was removed to read fluorescence (excitation 488 nm, emission 525 nm) using a Varioskan OD reader from Thermo Scientific (Waltham, MA, USA). Preliminary kinetic studies for EC monolayers’ permeability using VEGFA as a positive control showed that VEGA induced an increase in EC monolayer permeability at 24 h post cell exposure to VEGFA. Therefore, in most permeability assays, we monitored changes in EC monolayers’ permeability 24 h post exposure to the factors under study. Where indicated, we used an anti-human-PROS1 neutralizing monoclonal antibody and, as a control, a normal mouse IgG (Table 1). The effectiveness of the anti-PROS1 antibody used in neutralizing PROS1 activity has been confirmed by several previous studies [11,14]. Additional experiments in which inserts were either without dextran or without cells, respectively, were used to control for basal fluorescence levels or the maximal permeability of the filters, respectively. For permeability assays, data represent normalized values obtained from 3 to 6 independent experiments, each performed in triplicate, are expressed as percentage of control (diluent) ± SEM.

### 2.4. VEC Internalization Assay

This assay was carried out as described previously [35]. HUVECs were seeded onto fibronectin (5 μg/mL)-coated Nunc™ Lab-Tek™ chamber slides (Thermo Fisher; Waltham, MA, USA) and cultured for 24 h at 37 °C. Cells were then incubated with an anti-VEC clone BV6 monoclonal mouse antibody, which is directed against the N-terminal extracellular domain of VEC (Table 1) for 1 h at room temperature. Cells were then washed with PBS (Phosphate Buffer Saline) and exposed to either human PROS1 (10 µg/mL) or diluent in basal medium as indicated for either 30 min or 1 h. Cells were either directly fixed in 4% paraformaldehyde in PBS for membrane protein labeling or were acid washed for 15 min in cold 0.1 M glycine pH 2.0 in PBS and then fixed for labeling of internalized VEC. The acid-resistant staining corresponds to the internalized VEC fraction. Following fixation, cells were permeabilized in 0.1% Triton in PBS for 5 min and blocked with 0.5% BSA (Bovine Serum Albumin) in PBS for 30 min. A mix of biotinylated anti-mouse and rabbit antibodies and Fluorescein Avidin (all from Vector Laboratories; Peterborough, UK) were used as secondary antibodies/reagents (Table 1). Confocal microscopy acquisitions were performed on a confocal FV-1000 station installed on an inverted microscope IX-81 (Olympus). All micrographs are representative of 3 independent experiments and for each micrograph, 10–12 fields were imaged. The number of particles (internalized VEC vesicles) greater than 1 × 10^6^ pixel^2h^ were determined using Image v.J2 software. DAPI colored nuclei provided a count of total cell numbers. The ratio of the number of particles/numbers of cells are expressed as the mean ± SEM of three independent experiments. 

### 2.5. VEC Cleavage Assay

This assay was carried out as described previously [43]. Briefly, HUVECs were seeded at 10^5^ cells/well in 6 cm diameter dishes and grown for 3 days in EGM-2. Cells were then switched to EBM-2 for 24 h and exposed to either diluent or human PROS1 (10 µg/mL) for 24 h. Cell lysates were analyzed by SDS-PAGE on 10% polyacrylamide gels and Western blotting (20–30 μg of total protein lysate/lane) using anti-human VEC mouse antibody (BD Biosciences; Franklin Lakes, NJ, USA) directed against the C-terminal intracellular domain of VEC. For the measurement of protein content, we used the DC protein assay (Bio-Rad Laboratories Inc., Hercules, CA, USA) according to the manufacturer’s instructions. Conditioned media from untreated or human PROS1 stimulated cells (normalized to cell numbers) were collected, centrifuged to discard floating cells, and concentrated on ultra-centrifugal filter units (10 kDa cut-off) (Millipore Burlington, MA, USA) (Table 1). A volume of 20 to 30 μL of concentrated medium of each condition was analyzed by SDS-PAGE and Western blotting using the anti-VEC clone BV6 monoclonal mouse antibody (Table 1).

### 2.6. Western Blotting Assay

Western blotting assays were performed as described previously [11]. HUVECs were grown in 12-well plates at a density of 10^5^ cells/well in EGM-2 medium, for 3 days, then switched to EBM-2 for 24 h and exposed to human PROS1. In some experiments, cells were incubated with the indicated neutralizing antibody for 1 h prior to their exposure to either diluent or PROS1 (10 µg/mL). Cells were collected by scraping and were then lysed. Protein concentrations were determined using the DC protein assay (Bio-Rad Laboratories Inc., Hercules, CA, USA) according to the manufacturer’s instructions prior to equal amounts of proteins solubilized in Laemmli buffer being analyzed by SDS-PAGE and transferred onto nitrocellulose membranes using the iBlot system from Invitrogen (Thermo Fisher; Waltham, MA, USA). Membranes were probed with the following rabbit primary and secondary antibodies: anti-β-actin HRP (Horseradish Peroxidase) conjugate (clone 13E5), anti-phospho-HSP27 (Ser82), anti-HSP27 (clone D6W5V), anti-phospho-MLC2 (Thr18/Ser19), anti-MLC2 (clone D18E2), anti-phospho-c-Src (Tyr416), anti-c-Src, anti-phospho-PAK-1-Ser144/PAK-2-Ser141, anti-PAK-1, and mouse primary antibody: anti-human VEC, polyclonal antibodies directed either against phospho-VEC Ser^665^ or phospho-VEC Tyr^685^ (Table 1). Immunodetection was performed using chemiluminescent substrate ECL Plus (Amersham, Little Chalfont, UK) and an LAS-3000 imaging system (Fujifilm, Bedford, UK). Intensity of bands was quantified using Image v.J2 software. 

### 2.7. Actin and VEC Immunostaining

HUVECs were cultured at 7500 cells/well in fibronectin-coated Nunc™ Lab-Tek™ chamber Slide (Thermo Fisher; Waltham, MA, USA) for 24 h. Cells were switched to EBM-2 and exposed to the indicated factors for the specified durations. Cells were then fixed in 4% paraformaldehyde in PBS, permeabilized in 0.1% Triton X-100 in PBS and incubated with anti-VEC monoclonal antibody (BD Biosciences; Franklin Lakes, NJ, USA) (Table 1) followed by sequential incubations with biotin-labelled anti-mouse/anti-rabbit immunoglobulin G (Table 1) and fluorescein isothiocyanate (FITC)-labelled avidin D (Vector Laboratories), the latter was added together with 4 U/mL Texas Red–Phalloidin (Invitrogen; Thermo Fisher; Waltham, MA, USA) for simultaneous staining of F-actin. Slides were mounted in Vectashield with DAPI (Vector Laboratories; Peterborough, UK). Confocal microscopy acquisitions were performed on a confocal FV-1000 station installed on an inverted microscope IX-81 (Olympus, Tokyo, Japan).

### 2.8. Determination of Length of VEC Membrane Stretches

Ten fields of view, each containing between 120 and 200 cells, were analyzed using the Fiji software v.2.9.0. Auto-thresholding was carried out on VEC images using the Renyi Entropy method and the resulting images were skeletonized. Skeletons were then analyzed and total length of branches per image was calculated. In parallel, nuclear images were binarized following manual thresholding, a watershed was applied to separate adjacent nuclei, and the number of nuclei was determined using the Analyze Particles function. The total length of branches per image was divided by the number of nuclei to determine the average length of branches per cell. Student’s *t*-test was applied for statistical comparison of the conditions.

### 2.9. Determination of the Intracellular Fraction of VEC

Fifty cells per condition were analyzed using the Fiji software v.2.9.0 by a technician blinded to the experimental conditions. Two boundaries were drawn to encompass randomly selected cells per field, one encompassing the entire cell (including the membrane) and the second encompassing the intracellular space (excluding the membrane). The fluorescence intensity within the two boundaries was then measured and the signal from the inner boundary divided by that of the outer boundary. Student’s *t*-test was applied for statistical comparison of the conditions.

### 2.10. Statistical Analysis

Data obtained from at least 3 independent experiments were expressed either as mean values or percentages of control values ± SD or SEM. Western blot bands were analyzed by densitometry using Image J 1.49 V software and data are expressed in arbitrary units. Student’s test was used to compare the means of data from two experimental groups and one-way ANOVA was used when three or more experimental groups were compared using GraphPad Prism v.5 software. For all tests, *p*-values ≤ 0.05 were considered significant.

## 3. Results

### 3.1. Human PROS1 Increases Endothelial Cell Monolayer Permeability and Activates the p38 MAPK and Rho/ROCK Pathways

**Figure 1:**
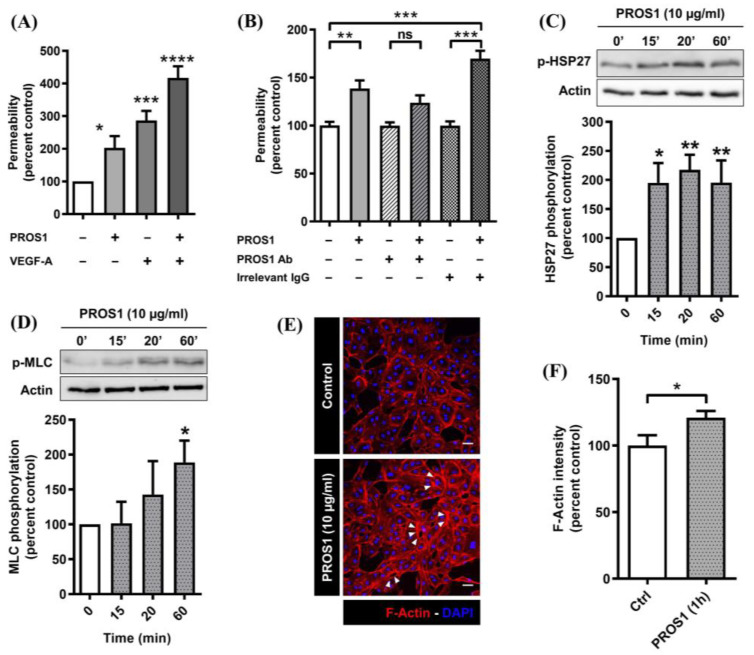
Human PROS1 increases endothelial monolayer permeability and activates the p38 MAPK and Rho/ROCK pathways. (**A**) Fold increase in EC monolayer permeability over control (diluent) induced by exposing ECs to either human PROS1 (10 µg/mL, 24 h) or VEGFA (20 ng/mL, 1 h) or a combination of human PROS1 (10 µg/mL, 24 h) followed by VEGFA (20 ng/mL, 1 h). For permeability assay, ECs plated on filter inserts were switched to EBM-2 and exposed to PROS1 and or VEGFA. 40 kDa FITC-dextran (1 mg/mL) was then added to the upper filter chamber for 30 min then 100 μL of the culture media of the lower chamber were removed, fluorescence was read (excitation 488 nm, emission 525 nm), and results are expressed as percentage of control (diluent) in fluorescence. (**B**) Permeability was assessed as in (**A**); results are expressed as percentage of control (vehicle) in EC layer permeability induced by exposing cells to either human PROS1 (10 µg/mL, 24 h) or a combination of human PROS1 (10 µg/mL, 24 h) and anti-human PROS1 IgG (10 µg/mL, 24 h) or human PROS1 (10 µg/mL, 24 h) and an irrelevant non-immune IgG (10 µg/mL, 24 h). Data in (**A**,**B**) are from 6 and 3 independent experiments, respectively, each performed in triplicate, and are expressed as percentage of control (diluent) ± SEM. **** *p* < 0.0001, *** *p* < 0.001, ** *p* < 0.01, ns indicates not significant. (**C**,**D**) Western blot analysis of HSP27 and MLC phosphorylation in cultured ECs. Subconfluent EC cultures were switched to EBM-2 plus PROS1 (10 μg/mL) or diluent for 15, 20, or 60 min. Cells were then lysed, and equivalent amounts of protein were analyzed for phospho-HSP27 or phospho-MLC2; band intensities were quantified and are represented as a percentage of the ratio of, respectively, phosphorylated HSP27 and MLC over actin. Data are from 6 and 4 independent experiments, respectively, each performed in triplicate, and expressed as percentage of control ± SEM. Statistical analyzes were performed using a Mann–Whitney test in order to compare each treatment time to the control ** *p* < 0.01, * *p* < 0.05. (**E**) Subconfluent ECs cultured on fibronectin-coated slides were exposed to either human PROS1 (10 μg/mL) or diluent for 1 h in EBM-2 medium. Cells were fixed, permeabilized and stained with Texas-Red phalloidin and DAPI. Slides were then analyzed by confocal microscopy. Arrows indicate changes in actin staining intensity, and white scale bars represents 50 µm length. (**F**) Quantification of actin staining intensity by Image v.J2 software. Results are presented as the ratio of the F-actin intensity/cell numbers and are expressed as the mean ± SEM of three independent experiments. Statistical analysis was performed using Prism 4.2 software.

In human plasma, the free and active forms of human PROS1 (~10 µg /mL) represent about 40% of the total circulating PROS1 (~25 µg/mL). Therefore, the experiments were performed using human PROS1 at 10 µg/mL which corresponds to the circulating physiological concentration of this protein. We have previously shown that, at this concentration, PROS1 does not induce cell death or exhibit any toxic effect on cultured endothelial cells [11]. Figure 1A shows that human PROS1 significantly increased EC monolayer permeability (2.03 ± 0.36 fold), to an extent similar (2.87 ± 0.29 fold) to VEGFA, a known endothelial permeability factor [35,38]. The increase in permeability appeared to be additive when monolayers were simultaneously exposed to both PROS1 and VEGFA (4.17 ± 0.36-fold), suggesting that this involved separate intracellular signaling pathways. As shown in Figure 1B, an anti-PROS1 antibody, but not a non-immune IgG from the same species used at the same concentration, reversed the effects of PROS1 on EC monolayer permeability.

To assess possible signaling events downstream of PROS1 that could be responsible for this permeability change, we next assessed the effect of human PROS1 on two important pathways associated with endothelial permeability: the p38 MAPK-HSP27 and the Rho/ROCK-MLC pathways. We observed that PROS1-induced HSP27 phosphorylation occurred within 15 min of PROS1 treatment and was sustained for over 1 h (Figure 1C). Similarly, the PROS1 treatment induced MLC phosphorylation (Figure 1D). Figure 1E,F show that this was associated with a substantial remodeling of actin filaments within ECs. Hence, our data suggest that human PROS1-induced endothelial permeability may be linked to the activation of p38 MAPK and Rho/ROCK pathways leading to the reorganization of actin filaments and cell contractility.

### 3.2. Human PROS1 Regulates Vascular Endothelial Cadherin (VEC) Internalization and Cleavage

**Figure 2:**
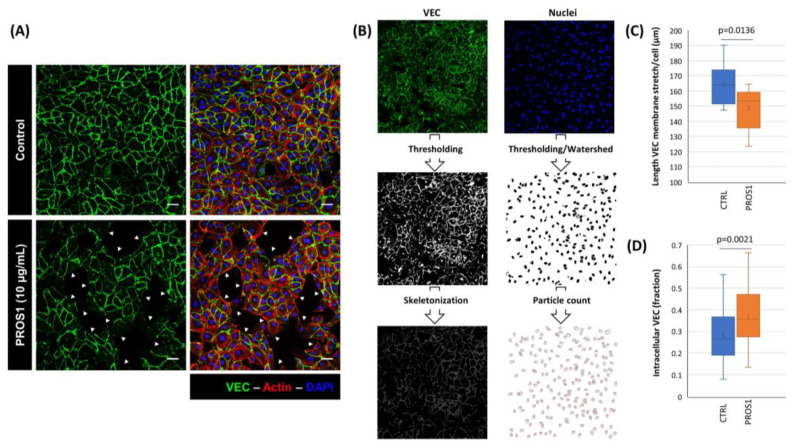
Human PROS1 stimulates VEC redistribution. (**A**) Representative micrographs, from 3 independent experiments, obtained by confocal microscopy depicting VEC staining in cultured ECs exposed to either diluent or human PROS1 (10 µg/mL) for 24 h. Cells were fixed, permeabilized and incubated with an anti-VEC monoclonal antibody and then with biotinylated anti-mouse followed by fluorescein avidin (green). Slides were mounted in vectashield with DAPI (blue) and analyzed by confocal microscopy. Arrows indicate examples of gap formation, and each white scale represents 50 µm length. (**B**) The VEC and nuclear images were analyzed using the Fiji software. The VEC image (**top left**) was subjected to auto-thresholding (**middle**) and skeletonized (**bottom**) prior to analysis of the skeletons’ branches properties. Thresholding was carried out on the nuclear images (**top right**) manually and they were binarized prior to the application of a watershed, and the nuclear count determined by the Particle Count function. (**C**) The total length of skeletonized branches was divided by the number of cells per field. n = 10 replicates were analyzed per condition. (**D**) Outer (including membrane) and inner (excluding membrane) cell boundaries were determined for 50 cells per condition and the ratio of inner to outer fluorescence intensity was determined. (**C**,**D**) Data shown as box-and-whiskers plot, with box representing 75% and whiskers 12.5% each of cells. Statistical analysis: Student *t*-test.

Since VEC is central to regulating vascular permeability, we next assessed whether human PROS1 affects VEC functioning. This revealed that exposing endothelial cells to PROS1 induced the redistribution of VEC at cell–cell contacts with an increase in the number of gaps between adjacent cells (Figure 2A–C). Indeed, skeletonization of images for VEC membrane staining (Figure 2B) revealed that treatment with PROS1 decreased the average length of these VEC contacts per cell (Figure 2C). In addition, a comparison of the ratio of intracellular to total cellular intensity for VEC staining revealed that PROS1 treatment significantly increased the intracellular fraction of VEC (Figure 2D). These PROS1-induced changes in VEC distribution may be linked to VEC phosphorylation, modifying the localization of the protein and disrupting cell–cell junctions.

**Figure 3:**
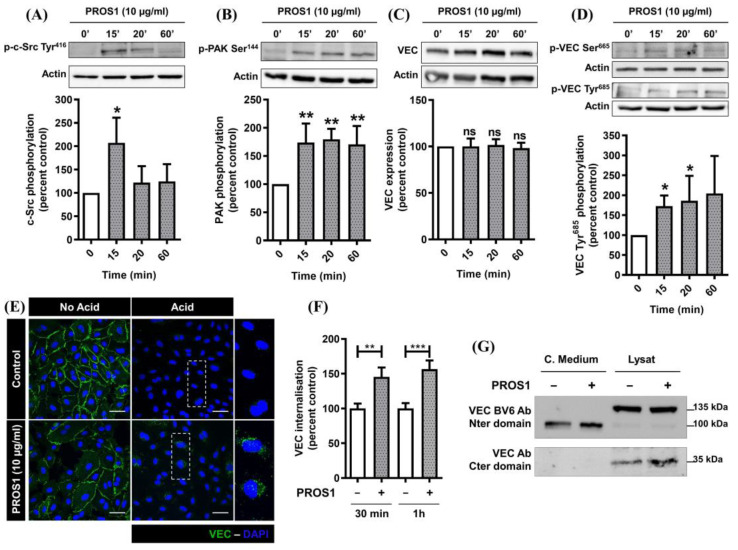
Human PROS1 promotes VEC phosphorylation, internalization, and cleavage. (**A**–**D**) Western blot analysis of Src and PAK-1 phosphorylation on, respectively, Tyr^416^ and Ser^144^ and VEC expression and phosphorylation on either Ser^665^ or Tyr^685^ residues. Subconfluent ECs grown in EGM-2 were switched to EBM-2 medium and exposed to either diluent or human PROS1 (10 µg/mL) for 15, 20, and 60 min. Cells were lysed, and equivalent amounts of protein from each sample were analyzed by western blotting for phospho-Tyr^416^ c-Src, phospho-Ser^144^ PAK-1, phospho-Ser^665^ VEC, phospho-Tyr^685^ VEC, or for total VEC using an anti-C-terminal VEC antibody. Bands’ intensities were quantified and normalized to that of actin. Data are from 5–7 independent experiments and expressed as percentage of control ± SEM. Statistical analysis were performed with a Mann–Whitney test in order to compare each treatment time to the control *** *p* < 0.001, ** *p* < 0.01, * *p* < 0.05. (**E**) VEC internalization experiments in which confluent cultured ECs grown in EGM-2 were switched to EBM-2, incubated with an anti-human N-terminal VEC antibody for 1 h, washed with EBM-2, and then exposed to either diluent or human PROS1 (10 µg/mL) for 1 h. Cells were either directly fixed for membrane protein labeling or acid washed to remove any membrane-bound antibody and then fixed. Both preparations (without or with acid wash) were permeabilized and incubated with a mix of horse biotinylated anti-mouse and anti-rabbit antibodies followed by Fluorescein Avidin. Confocal microscopy acquisitions were performed on a confocal FV-1000 station installed on an inverted microscope IX-81 (Olympus); each white scale represents 50 µm length. (**F**) Increase in VEC internalization compared to control estimated by analyzing number of cytoplasmic particles per cell after exposure to human PROS1 (10 µg/mL) for either 30 min or 1 h. Data were obtained from 3 independent experiments each. For each independent experiment, 10–12 microscopic fields were counted, and data are expressed as percentage of control ± SEM. *** *p* < 0.0001, ** *p* < 0.01. (**G**) VEC cleavage experiments in which subconfluent ECs grown in EGM-2 were switched to EBM-2 and exposed to human PROS1 (10 µg/mL) for 24 h. Cell-conditioned media were collected and concentrated and cells were lysed. Equivalent amounts of proteins of either concentrated cell conditioned media (normalized to total protein) and cell lysates were resolved by SDS-PAGE, transferred to a nitrocellulose membrane, and probed with either anti N-terminal- or C-terminal-specific VEC antibodies. Data were obtained from 3 independent experiments, each performed in duplicate.

We therefore investigated the effect of PROS1 treatment on VEC phosphorylation. We found that PROS1 treatment induced a rapid and transient phosphorylation of c-Src protein on Tyr^416^ (Figure 3A), of PAK-1 kinase on Ser^144^ (Figure 3B), and of their corresponding target sites on VEC, Tyr^685^ and Ser^665^ (Figure 3D). Moreover, using an anti C-terminal VEC antibody, we showed that the exposure of cells to PROS1 for 15 to 60 min did not result in significant changes in VEC expression levels (Figure 3C). The induction of PAK-1 autophosphorylation and the subsequent phosphorylation of VEC on Ser^665^ by VEGFA are associated with VEC internalization [34,35,36,37]. Also, VEGFA-induced phosphorylation of c-Src on Tyr^416^ and subsequently that of VEC on Tyr^685^ has been reported to trigger VEC cleavage into both a 90–100 kDa N-terminal fragment that is released into the medium and a 35 kDa C-terminal fragment that remains intracellular [38,39,40]. Therefore, we next investigated the effects of PROS1 on the induction of VEC internalization and cleavage.

Reminiscent of the results shown in Figure 2D, 30 min to 1 h treatment with PROS1 PROS1 induced a significant increase in VEC internalization (Figure 3E,F). Also, cell exposure to PROS1 for 24 h led to increased levels of both 100 kDa and 35 kDa VEC fragments in cell-conditioned media and cell lysates, respectively. Hence, PROS1 treatment appears to phenocopy the effects of VEGFA on VEC cellular distribution and cleavage.

### 3.3. Implication of Both MERTK and TYRO3 Receptors in PROS1-Induced c-Src and VEC Phosphorylation

PROS1 is a ligand for TYRO3 and MERTK, two members of the TAM family [6]. Through the inhibition of TAMs by siRNA-mediated silencing and neutralizing antibodies, we previously reported that the effects of PROS1 on angiogenesis are mainly mediated by MERTK [11]. In the present study, the use of siRNAs targeting MERTK or neutralizing antibodies in endothelial monolayer permeability assays was complicated by both the duration of those experiments (over 5 days) and our observation that, even in the absence of PROS1 treatment, MERTK silencing or neutralizing already drastically increased endothelial layer permeability. Instead, since an analysis of either VEC or c-Src phosphorylation can be performed within minutes, we used anti-TAM neutralizing antibodies, as previously shown [11,16], to identify the TAM receptors involved in these effects.

**Figure 4:**
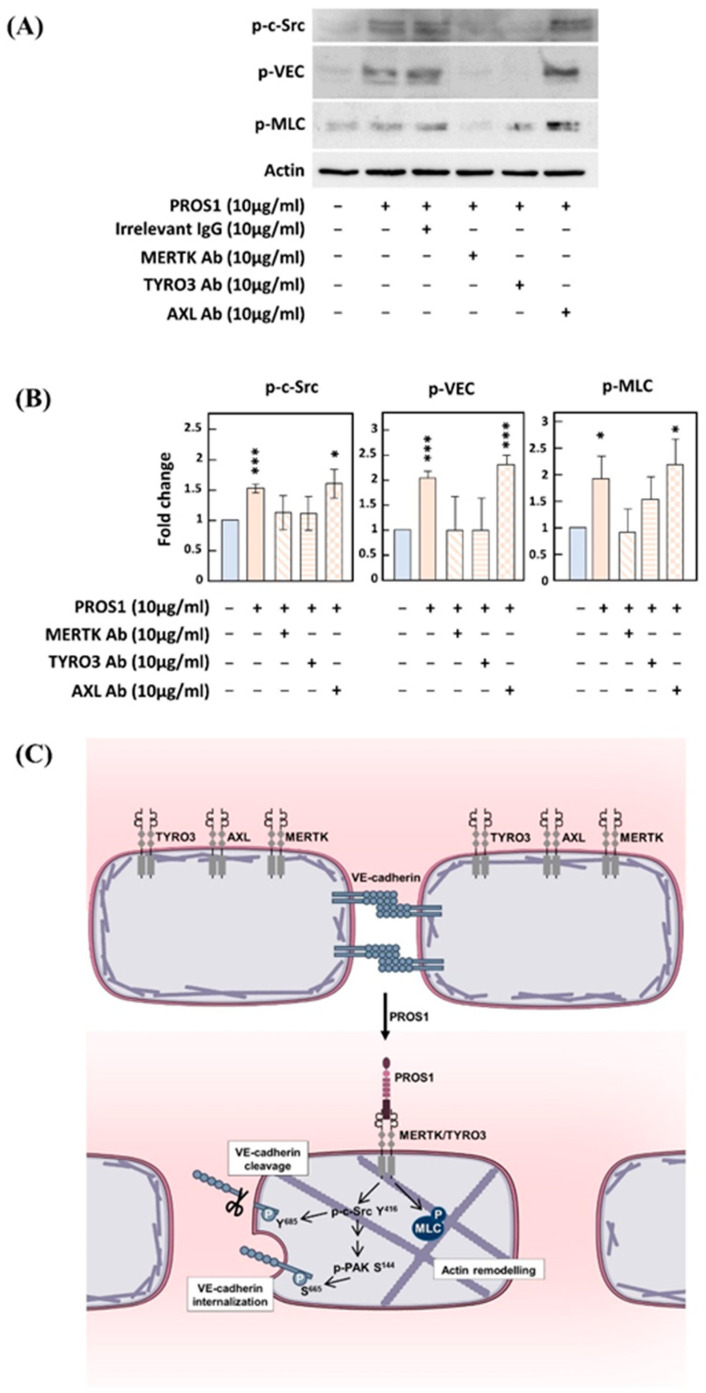
Implication of both MERTK and TYRO3 receptors in PROS1-induced Src and VEC phosphorylation. (**A**,**B**) represent Western blot analysis (**A**) and subsequent quantification (**B**) of VEC phosphorylation on Tyr^685^, c-Src phosphorylation on Tyr^416^ or MLC phosphorylation. (**B**) Integrated intensity for the target proteins was divided by that for actin, and all conditions were normalized to their corresponding untreated controls. Analysis was performed with the Fiji software. (**C**) Proposed mechanism through which PROS1 interferes with EC permeability. PROS1 activates its tyrosine kinase receptors MERTK and TYRO3, leading to the phosphorylation of c- Src on Tyr^416^ and PAK-1 on Ser^144^ which in turn phosphorylate, respectively, VEC at Tyr^685^ and Ser^665^, two specific sites involved in the cleavage and internalization of VEC, respectively. The PROS1/MERTK-TYRO3/c-Src-Tyr^416^/VEC-Tyr^685^ pathway activates the cleavage of VEC extracellular domain, which destabilizes cell–cell junctions. The PROS1/MERTK-TYRO3/ PAK-1-Ser^144^/VEC-Ser^665^ pathway activates VEC internalization thus preventing VEC recognition between two adjacent endothelial cells. The Rho/ROCK/MLC pathway involved in the regulation of endothelial permeability is also activated by human PROS1. * *p* < 0.05; *** *p* < 0.001.

Figure 4A shows that TYRO3- and MERTK- but not AXL-neutralizing antibodies inhibited the induction of both VEC phosphorylation on Tyr^685^ and c-Src phosphorylation on Tyr^416^ by PROS1. Similarly, the neutralization of TYRO3 or MERTK, but not AXL, inhibits PROS1-induced MLC phosphorylation (Figure 4B). These results suggest that both MERTK and TYRO3 mediate the effects of PROS1 on c-Src and VEC signaling as well as the activation of the Rho-ROCK pathway. Taken together, our data, summarized in Figure 4C, suggest that PROS1 interacts with both MERTK and TYRO3 to regulate endothelium permeability through the activation of c-Src and PAK-1, followed by the phosphorylation of VEC on the target sites for these two kinases. VEC phosphorylation on Tyr^685^ or Ser^665^ is then involved in VEC cleavage and internalization, respectively. VEC cleavage may lead to cell–cell junction destabilization while VEC internalization prevents adjacent cell–cell recognition. Furthermore, the MERTK and TYRO3 mediate the phosphorylation of MLC by Rho/ROCK and p38 MAPK pathways, possibly leading to actin cytoskeleton remodeling and the subsequent destabilization of cell junctions, with a resulting increase in endothelium permeability.

## 4. Discussion

In this report we provide evidence that PROS1 increases endothelial permeability in a TYRO3- and MERTK-dependent manner, activates the Rho/ROCK and p38 MAPK pathways, and triggers actin remodeling as well as VEC cleavage and/or internalization.

As depicted in Figure 1A, the increase in EC monolayer permeability was higher if the cells were exposed simultaneously to both PROS1 and VEGFA, implying that PROS1 at its physiological circulating concentration induces a substantial increase in both the basal and VEGFA-induced permeability of EC monolayer. Hence, circulating PROS1 is likely to contribute to the fine tuning of pro- versus anti-permeability factors, revealing a novel vascular function of this protein. While it is well documented that several endothelium-produced factors such as PROS1, TFPI (Tissue Factor Pathway Inhibitor), t-PA (tissue Plasminogen Activator), PAI-1 (Plasminogen Activator Inhibitor-1), and antithrombin III are major regulators of blood coagulation and hemostasis [44,45,46,47], little is known about how such circulating factors, to which the endothelium is permanently exposed, influence its functions in general and barrier function in particular. Since the endothelium produces over 40% of the circulating PROS1 [22] and expresses PROS1 TAM tyrosine kinase receptors [11] for this factor, it was important to assess the potential role of PROS1 on the biology of the endothelium.

The novel effect of PROS1 on endothelium permeability we describe in the present report is relevant for both vessel development and functional integrity. Saller et al. reported that PROS1-/- mice die in utero from a coagulopathy and associated intracranial hemorrhages [23]. The study by Burstyn-Cohen et al. confirmed that a PROS1 homozygous deficiency is lethal in mice at the embryonic stage. In mice, the major phenotype associated with either complete or conditional deletion of PROS1 in vascular smooth muscle cells was vascular dysgenesis resulting in vessel leakage and hemorrhage [22].

We also show that the exposure of cultured ECs to human PROS1 for one hour activates the Rho/ROCK pathway as deduced from the rapid phosphorylation of its substrate MLC. MLC phosphorylation is directly involved in EC actin rearrangement, cell body contraction, and gap formation [48,49]. Such an effect of PROS1 is similar to that reported for other vascular permeabilizing factors such as VEGFA, thrombin, and histamine [48,50]. Furthermore, the exposure of endothelial cells to human PROS1 induces the activation of the p38 MAPK pathway within 15 min as suggested by the sharp phosphorylation of HSP27, a substrate for this MAPK. It is well documented that the Rho GTPases Rac1 make the endothelial barrier more restrictive through the formation of lamellipodia, whereas RhoA increases barrier permeability through the formation of contraction-related stress fibers [51].

In the present report, we show that the exposure of ECs to PROS1 leads to c-Src activation as determined from the sharp phosphorylation of c-Src on Tyr^416^, suggesting prior dephosphorylation of Tyr^527^ by SHP-2. This is in agreement with our previous report which demonstrated that PROS1/TAM interaction leads to SHP-2 recruitment in ECs [11]. Therefore, through its ability to dephosphorylate either VEGFR2 (inhibiting its signaling) or c-Src (activating its signaling), SHP-2 seems to stand at the crossroads of mediating both PROS1 and VEGFA action on either vascular permeability or angiogenesis. Full activation of c-Src first requires its dephosphorylation by SHP-2 at the inhibitory Tyr^527^ site, followed by c-Src auto-phosphorylation at the stimulatory Tyr^416^ site, enabling further phosphorylation of downstream substrates such as VEC on Tyr^685^ [52,53,54,55].

We report that exposure of ECs to PROS1 induced both VEC phosphorylation on Tyr685 as well as the cleavage of its N-terminal domain and its release into the medium as a soluble 90–100 kDa form, referred to as sVEC. VEC cleavage at its N-terminal domain, leading to the release into the medium of a soluble 90–100 kDa form, is a process that has been described previously for VEGFA-mediated increased vascular permeability [38,39,40,56,57] and is revealed for the first time for human PROS1. Many proteases, such as ADAM 10 [32], ADAM 12 [33], MMP 2, and MMP 9 [31,57] have been documented to catalyze VEC N-terminal cleavage with the release of a 90–100 kDa soluble form. Our observed PROS1-induced VEC N-terminal cleavage may be linked to the ability of PROS1 to directly or indirectly activate one or several of these or different proteases.

Besides inducing VEC phosphorylation by c-Src, we also report here that human PROS1 induces PAK-1 phosphorylation on Ser^144^ with subsequent VEC phosphorylation on Ser^665^ and internalization. Such a mechanism is known to be responsible, at least in part, for VEGFA-induced increases in endothelial cell permeability [35], and is revealed for the first time here for human PROS1. It has also been shown that the phosphorylation of VEC on Ser^665^ leads to the dissociation of VEC from catenin and to its internalization, which in turn weakens adherent junctions and subsequently increases vascular permeability [30,34,35,36,37,56,58,59,60].

The use of neutralizing TAM receptor antibodies in Western blotting assays has led us to conclude that both MERTK and TYRO3 receptors are implicated in PROS1-induced VEC phosphorylation on Tyr^685^, c-Src protein phosphorylation on Tyr^416^, and MLC phosphorylation. In agreement with our findings, it has been documented that the TAM activation patterns caused by PROS1 and its structural homolog Gas6 (Growth Arrest Specific 6) are distinct: AXL is activated exclusively by Gas6 but not PROS1, while TYRO3 and MERTK can be activated by either Gas6 or PROS1 [7]. In ECs, it is well established that PROS1 may activate both MERTK and TYRO3 [8,11]. In a different experimental set-up, using cultured brain endothelial cells as a model for the brain–blood barrier, previous studies have reported the protective effects of PROS1 on the integrity of the blood–brain barrier involving either a MERTK- [61] or a TYRO3- [8] dependent mechanism. The main structures responsible for the barrier function are tight junctions which are the most represented in the endothelium of the blood–brain barrier [62]. In contrast, adherent junctions are more abundant in large vessels. For the tight junctions in the endothelium of the blood–brain barrier, Miner et al. [61] described MERTK-dependent regulatory PROS1 effects involving the remodeling of the actin cytoskeleton via the activation of Rac1. Zhu et al. [8] described that, in cultured brain endothelial cells, PROS1/ TYRO3 interaction leads to TYRO3’s association with S1P1 (Sphingosine 1-phosphate receptor 1), to Rac1 pathway activation, and to the formation of cortical actin rings, thereby reinforcing the vascular integrity of the blood–brain barrier.

In short, using cultured ECs we uncovered, for the first time, a novel mechanism by which PROS1, at its circulating levels, increases endothelial permeability. This newly described effect of PROS1 is both TYRO3- and MERTK-dependent, involves Rho/ROCK, p38 MAPK pathways activation, actin remodeling, and VEC phosphorylation on Ser^665^ and Tyr^685^, as well as its cleavage and/or its internalization. Alterations in VEC functioning are associated with several pathologies such as metastatic breast cancer [63]. Hence, our findings suggest that the involvement of the PROS1/TAM receptors axis in the metastatic process through its regulation of VEC internalization and cleavage should urgently be investigated.

## Figures and Tables

**Table 1:** Information relating to the antibodies used.

Target	Name/Catalog#	Supplier	Type of Antibody	Concentration
Primary antibodies				
Actin	β-actin (13E5) HRP conjugate/#5125	Cell Signaling Technology (Danvers, MA, USA)	Monoclonal Rabbit IgG	WB 1:1000
HSP27	HSP27 (D6W5V)/#95357	Cell Signaling Technology	Monoclonal Rabbit IgG	WB 1:1000
p-HSP27	Phospho-HSP27 (Ser82)/#2401	Cell Signaling Technology	Polyclonal Rabbit AB	WB 1:1000
MLC	Myosin Light Chain 2 (D18E2)/#8505	Cell Signaling Technology	Monoclonal Rabbit IgG	WB 1:1000
p-MLC	Phospho-Myosin Light Chain 2 (Thr18/Ser19)/#3674	Cell Signaling Technology	Polyclonal Rabbit AB	WB 1:1000
PAK	PAK1 Antibody/#2602	Cell Signaling Technology	Polyclonal Rabbit AB	WB 1:1000
p-PAK	Phospho-PAK1(Ser144)/PAK2 (Ser141)/#2606	Cell Signaling Technology	Polyclonal Rabbit AB	WB 1:1000
c-Src	Src Antibody/#2108	Cell Signaling Technology	Polyclonal Rabbit AB	WB 1:1000
p-c-Src	Phospho-Src Family (Tyr416)/#2101	Cell Signaling Technology	Polyclonal Rabbit AB	WB 1:1000
VEC	Purified Mouse Anti-Human CD144 (55-7H1)/#555661	BD Biosciences	Monoclonal Mouse IgG	WB 1:1000 IF 1:400
	Anti-VE-cadherin AB, (clone BV6) /#MABT134	Millipore	Monoclonal Mouse IgG	WB 1:1000
p-VEC	Phospho-VEC (Ser665)	*	Polyclonal Rabbit AB	WB 1:1000
	Phospho-VEC (Tyr 685)	**	Polyclonal Rabbit AB	WB 1:1000
Secondary antibodies				
-	Anti-Mouse IgG—Peroxidase AB /#A4416	Sigma-Aldrich (Burlington, MA, USA)	Polyclonal Goat IgG	WB 1:4000/1:5000
-	Anti-Rabbit IgG—Peroxidase AB /#A0545	Sigma-Aldrich	Polyclonal Goat IgG	WB 1:4000/1:5000
-	Anti-Mouse/Rabbit IgG Antibody (H+L), Biotinylated/#BA-1400	Vector Laboratories	Polyclonal Horse IgG	IF 1:100
Neutralizing and Irrelevant antibodies				
AXL	Human AXL Antibody/#AF154	R&D Systems (Minneapolis, MN, USA)	Polyclonal Goat IgG	10 µg/mL
MERTK	Human MERTK Antibody/#AF891	R&D Systems	Polyclonal Goat IgG	10 µg/mL
PROS1	Anti-Human Protein S /#A038401-2	Agilent Dako (Santa Clara, CA, USA)	Polyclonal Rabbit AB	10 µg/mL
TYRO3	Human Dtk Antibody/#AF859	R&D Systems	Polyclonal Goat IgG	10 µg/mL
-	Normal Rabbit IgG Control/#AB105C	R&D Systems	Polyclonal Rabbit IgG	10 µg/mL
-	Normal Goat IgG Control/#AB108C	R&D Systems	Polyclonal Goat IgG	10 µg/mL

*/** Polyclonal antibodies directed either against phospho-VEC Ser665 or phospho-VEC Tyr685 were kind gifts from, respectively, Dr Julie Gavard (Team SOAP, CRCI2NA, Nantes University, INSERM, CNRS, Nantes, France) and Dr Isabelle Vilgrain (Grenoble Alpes University, INSERM, CEA, BGE-Biomics, Grenoble, France).

## Data Availability

The raw data supporting the conclusions of this article will be made available by the authors on request.
